# Whole genome sequencing analyses of *Listeria monocytogenes* that persisted in a milkshake machine for a year and caused illnesses in Washington State

**DOI:** 10.1186/s12866-017-1043-1

**Published:** 2017-06-15

**Authors:** Zhen Li, Ailyn Pérez-Osorio, Yu Wang, Kaye Eckmann, William A. Glover, Marc W. Allard, Eric W. Brown, Yi Chen

**Affiliations:** 1 Washington State Department of Health, Public Health Laboratories, Shoreline, Washington, USA; 2 Center for Food Safety and Applied Nutrition, Food and Drug Administration, College Park, MD USA

**Keywords:** Listeriosis, Ice cream, Outbreak, Whole genome sequencing, Core genome multilocus sequence typing

## Abstract

**Background:**

In 2015, in addition to a United States multistate outbreak linked to contaminated ice cream, another outbreak linked to ice cream was reported in the Pacific Northwest of the United States. It was a hospital-acquired outbreak linked to milkshakes, made from contaminated ice cream mixes and milkshake maker, served to patients. Here we performed multiple analyses on isolates associated with this outbreak: pulsed-field gel electrophoresis (PFGE), whole genome single nucleotide polymorphism (SNP) analysis, species-specific core genome multilocus sequence typing (cgMLST), lineage-specific cgMLST and whole genome-specific MLST (wgsMLST)/outbreak-specific cgMLST. We also analyzed the prophages and virulence genes.

**Results:**

The outbreak isolates belonged to sequence type 1038, clonal complex 101, genetic lineage II. There were no pre-mature stop codons in *inlA*. Isolates contained *Listeria* Pathogenicity Island 1 and multiple internalins. PFGE and multiple whole genome sequencing (WGS) analyses all clustered together food, environmental and clinical isolates when compared to outgroup from the same clonal complex, which supported the finding that *L. monocytogenes* likely persisted in the soft serve ice cream/milkshake maker from November 2014 to November 2015 and caused 3 illnesses, and that the outbreak strain was transmitted between two ice cream production facilities. The whole genome SNP analysis, one of the two species-specific cgMLST, the lineage II-specific cgMLST and the wgsMLST/outbreak-specific cgMLST showed that *L. monocytogenes* cells persistent in the milkshake maker for a year formed a unique clade inside the outbreak cluster. This clustering was consistent with the cleaning practice after the outbreak was initially recognized in late 2014 and early 2015. Putative prophages were conserved among prophage-containing isolates. The loss of a putative prophage in two isolates resulted in the loss of the *Asc*I restriction site in the prophage, which contributed to their *Asc*I-PFGE banding pattern differences from other isolates.

**Conclusions:**

The high resolution of WGS analyses allowed the differentiation of epidemiologically unrelated isolates, as well as the elucidation of the microevolution and persistence of isolates within the scope of one outbreak. We applied a wgsMLST scheme which is essentially the outbreak-specific cgMLST. This scheme can be combined with lineage-specific cgMLST and species-specific cgMLST to maximize the resolution of WGS.

## Background

*Listeria monocytogenes* is a Gram-positive, facultative intracellular bacterium that causes high mortality foodborne illnesses through contaminated food products [1]. *L. monocytogenes* exists in different environments due to its hardiness in harsh conditions, such as a wide pH range, high salt concentrations and ability to grow and persist at refrigeration temperatures [2]. These unique characteristics have made *L. monocytogenes* one of the major threats to the food industry and public health. Several listeriosis outbreaks occurred in United States recently, linked to dairy products and fresh produce [3–5]. Ice cream-associated outbreaks are rarely reported. However, two epidemiologically unrelated outbreaks were linked to contaminated ice cream in recent years. A 2010–2015 multistate listeriosis outbreak was linked to contaminated ice cream manufactured in the southern United States [6]. In late 2014, a different listeriosis outbreak in Washington State, unrelated to the 2010–2015 multistate outbreak, occurred in a hospital (Hospital X) in the Pacific Northwest of the United States, involving patients hospitalized for other medical conditions prior to exposure to milkshakes made from contaminated ice cream mixes manufactured in a company (Company A) [7]. Following the investigation of the Washington State outbreak, intensive cleaning and sanitizing were conducted in the facility and hospital kitchen, although cleaning of the soft serve shake freezer took extra efforts, because milkshake was made inside the machine and disassembly was required for thorough cleaning [7]. In November 2015, another patient from Hospital X, hospitalized for other conditions prior to exposure to *L. monocytogenes*, was linked to contaminated milkshakes by pulsed-field gel electrophoresis (PFGE) [8]. Hospital X was using a different brand of ice cream mix from the 2014 outbreak, which was tested negative for *L. monocytogenes*; but isolates recovered from the milkshake samples and swab samples from the milkshake machine matched the outbreak-associated isolates collected in 2014 [8], confirming that this third patient was also associated with this outbreak.

Single nucleotide polymorphism (SNP)-based and multilocus sequence typing (MLST) allele-based whole genome sequence (WGS) analyses have been utilized to support the findings of the listeriosis outbreak investigations and offer various advantages over PFGE [5, 6, 9]. SNP-based analyses could target SNPs in the whole genome (i.e. entire genome including coding and noncoding regions) or the core genome (i.e. coding regions that are present in a set of strains). A whole genome MLST (wgMLST) scheme, targeting a specific pan-genome of 4797 loci defined based on over 150 publicly available reference genomes of *L. monocytogenes* [10], was implemented in PulseNet [9]. An alternative way to perform whole genome-based MLST is to target the entire coding loci that are specific to a set of closely-related isolates (e.g, those of the same outbreak strain). This scheme may target loci unique to these isolates, which are not included in any pre-defined pan-genome locus set.

Four *L. monocytogenes* species-specific core genome MLST (cgMLST) schemes have been developed [4, 11–13]. Further, lineage-specific cgMLST schemes for 3 genetic lineages of *L. monocytogenes* were developed to improve the discriminatory power [4]. The objective of this study is to determine whether the results of whole genome SNP analysis, whole genome-specific MLST (wgsMLST)/outbreak-specific cgMLST, lineage-specific and species-specific cgMLST analyses were consistent with PFGE, and could support epidemiological evidence to delineate the Hospital X - acquired outbreak.

## Results

Isolates selected for WGS analysis are listed in Table 1. The outbreak-associated isolates had sequence type (ST) 1038, belonging to clonal complex (CC) 101, a genetic lineage II clonal group [4]. The non-outbreak isolate CFSAN028854 (as discussed below) had ST5, which belonged to CC5, a serotype 1/2b or 3b clonal group [4], and thus it is not illustrated in the phylogenetic trees. The outbreak isolates contained *Listeria* pathogenicity island (LIPI)-1, internalin A, B, C, E, F, H, J and P. They did not contain LIPI-3 or LIPI-4. There were no premature stop codons (PMSC) in *inlA*.

**Table 1:** List of *L. monocytogenes* isolates analyzed in this study

Isolate ID	Collection time	Source Type	*Asc*I-PFGE pattern	*Apa*I-PFGE pattern	SRA ID or GenBank Accession
PNUSAL001207	November, 2014	Clinical	*Asc*I-P1	*Apa*I-P1	SRR1745448
PNUSAL001241	December, 2014	Clinical	*Asc*I-P2	*Apa*I-P1	SRR1745474
CFSAN028842	December, 2014	Ice cream/Hospital X	*Asc*I-P1	*Apa*I-P1	SRR3130313
CFSAN028843	December, 2014	Ice cream/Hospital X	*Asc*I-P1	*Apa*I-P1	SRR3091402
CFSAN028844	December, 2014	Ice cream/Hospital X	*Asc*I-P1	*Apa*I-P1	SRR3130327
CFSAN028845	December, 2014	Ice cream/Hospital X	*Asc*I-P1	*Apa*I-P1	SRR3066080
CFSAN028846	December, 2014	Ice cream/Hospital X	*Asc*I-P1	*Apa*I-P1	SRR3130329
CFSAN028847	December, 2014	Ice cream/Hospital X	*Asc*I-P1	*Apa*I-P1	SRR3091403
CFSAN028848	December, 2014	Ice cream/Company A	*Asc*I-P1	*Apa*I-P1	SRR3091404
CFSAN028849	December, 2014	Ice cream/Company A	*Asc*I-P1	*Apa*I-P1	SRR3091405
CFSAN028850	December, 2014	Ice cream/Company A	*Asc*I-P1	*Apa*I-P1	SRR3130331
CFSAN028851	December, 2014	Ice cream/Company A	*Asc*I-P1	*Apa*I-P1	SRR3091406
CFSAN028852	December, 2014	Environmental/Company A	*Asc*I-P1	*Apa*I-P1	SRR3130333
CFSAN028853	December, 2014	Environmental/Company A	*Asc*I-P1	*Apa*I-P1	SRR3130335, MAKW00000000.1
CFSAN028855	December, 2014	Environmental/Company A	*Asc*I-P1	*Apa*I-P1	SRR3130404
CFSAN028856	December, 2014	Environmental/Company A	*Asc*I-P1	*Apa*I-P1	SRR3130406
CFSAN028857	December, 2014	Environmental/Company A	*Asc*I-P3	*Apa*I-P1	SRR3130409
CFSAN028858	December, 2014	Environmental/Company A	*Asc*I-P1	*Apa*I-P1	SRR3130413
CFSAN028859	December, 2014	Environmental/Company A	*Asc*I-P1	*Apa*I-P1	SRR3130415
CFSAN028860	December, 2014	Environmental/Company A	*Asc*I-P1	*Apa*I-P1	SRR3130350
CFSAN028861	December, 2014	Environmental/Company A	*Asc*I-P2	*Apa*I-P1	SRR3130375
CFSAN029502	December, 2014	Environmental/Company A	*Asc*I-P1	*Apa*I-P1	SRR3130341
CFSAN030692	March, 2015	Environmental/Company A	*Asc*I-P1	*Apa*I-P1	SRR1974103
CFSAN032836	April, 2015	Environmental/Company B	*Asc*I-P1	*Apa*I-P1	SRR2035442
CFSAN043359	November, 2015	Ice cream/Hospital X	*Asc*I-P1	*Apa*I-P1	SRR3052035
CFSAN043360	November, 2015	Ice cream/Hospital X	*Asc*I-P1	*Apa*I-P1	SRR3053137
CFSAN043361	November, 2015	Ice cream/Hospital X	*Asc*I-P1	*Apa*I-P1	SRR3086932
CFSAN043362	November, 2015	Environmental/Hospital X	*Asc*I-P1	*Apa*I-P1	SRR3086935
CFSAN043363	November, 2015	Environmental/Hospital X	*Asc*I-P1	*Apa*I-P1	SRR3086936
CFSAN043364	November, 2015	Environmental/Hospital X	*Asc*I-P1	*Apa*I-P1	SRR3052036
PNUSAL001911	November, 2015	Clinical	*Asc*I-P1	*Apa*I-P1	SRR2994642
CFSAN028854	December, 2014	Environmental/Company A	*Asc*I-P4	*Apa*I-P2	SRR3130337
CFSAN004336	NA	Food	NA	NA	SRR1818032

**Fig. 1:**
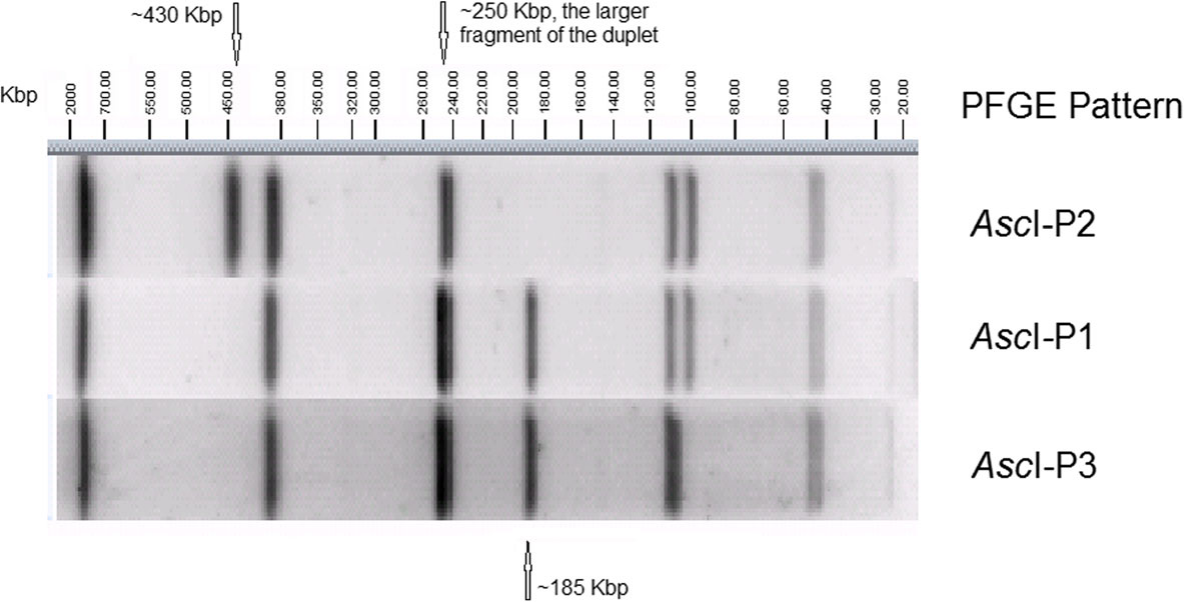
Three *Asc*I-PFGE banding patterns observed among outbreak-associated isolates

The two clinical isolates collected in November and December 2014 exhibited two PFGE profiles, *Asc*I-P1/*Apa*I-P1 and *Asc*I-P2/*Apa*I-P1 (Fig. 1). Isolates from ice cream products manufactured by Company A and environmental samples from Company A facility areas, and isolates of unopened ice cream products and machine-dispensed products from Hospital X, collected after the outbreak recognition in 2014, exhibited *Asc*I-P1/*Apa*I-P1, *Asc*I-P2/*Apa*I-P1, *Asc*I-P3/*Apa*I-P1 and *Asc*I-P4/*Apa*I-P2. One environmental isolate was collected in March 2015 from Company A and one environmental isolate was collected in April 2015 from Company B who purchased dairy ingredients from Company A; and they both exhibited *Asc*I-P1/*Apa*I-P1. After the identification of the case-patient in November 2015, isolates were collected from the ice cream that remained in and were dispensed from the milkshake maker in Hospital X and environmental samples from different areas (e.g., side walls of internal parts and nozzle assembly) of the milkshake maker; and they exhibited *Asc*I-P1/*Apa*I-P1, the same as the 2015 clinical isolate. *Asc*I-P1/*Apa*I-P1, *Asc*I-P2/*Apa*I-P1, *Asc*I-P3/*Apa*I-P1 were rare PFGE profiles in PulseNet; prior to this outbreak only one isolate in 2010, with no epidemiological link to this outbreak, exhibited *Asc*I-P2/*Apa*I-P1. Overall, 27 of 29 food and environmental isolates had indistinguishable PFGE profiles from the 3 clinical isolates. Two other environmental isolates had PFGE profiles not observed in any clinical isolates.

**Fig. 2:**
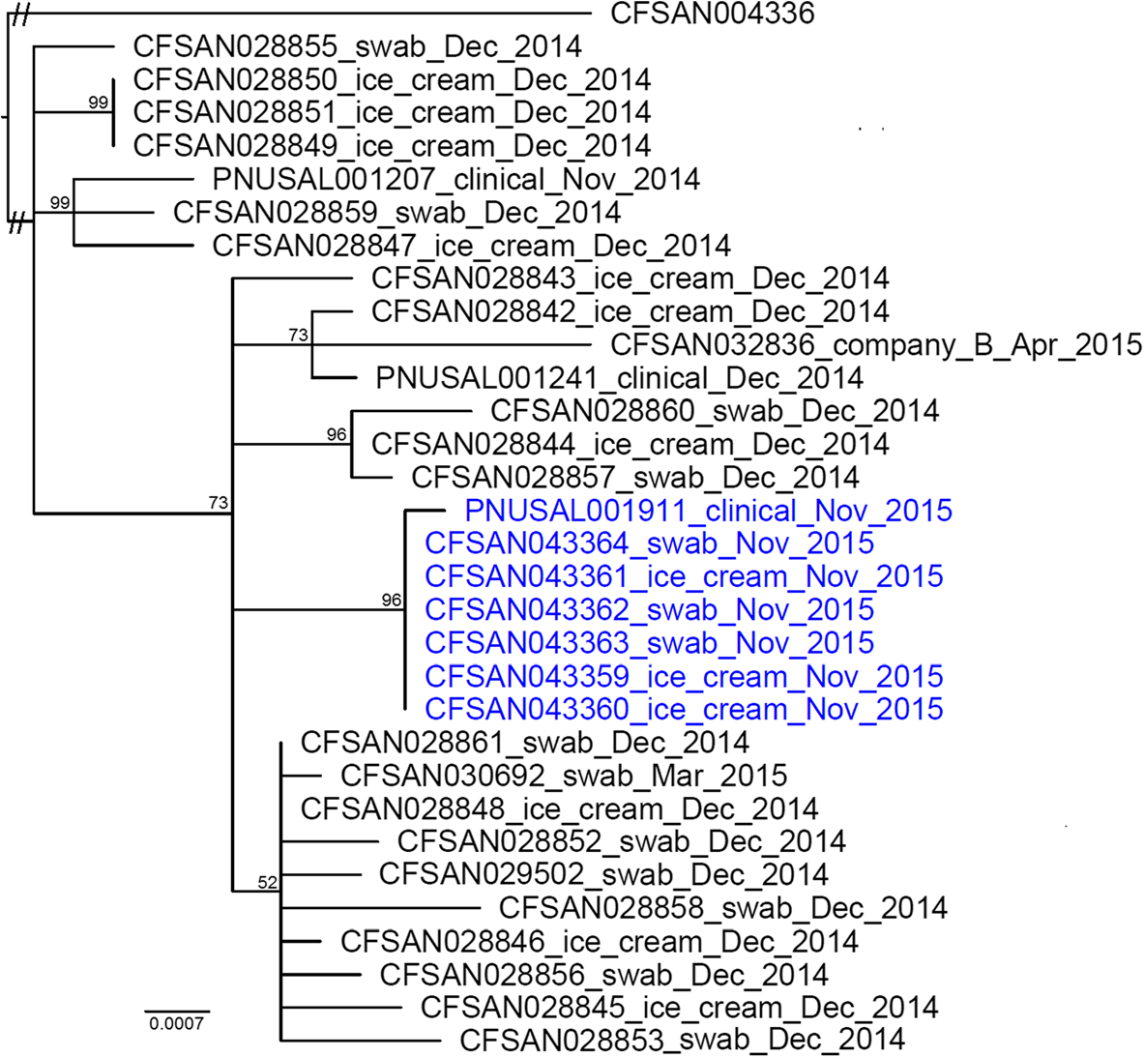
Maximum likelihood phylogeny of outbreak-associated isolates based on single nucleotide polymorphisms (SNPs) identified by the Center for Food Safety and Applied Nutrition (CFSAN) SNP Pipeline using CFSAN004336 for comparison. The tree is rooted at midpoint. Isolate ID is followed by sample type and collection year and abbreviation of month. The isolates that persisted in the milkshake maker until November 2015 and the clinical isolate collected in November 2015 are in *blue color*. The isolates collected in March and April 2015 were from ice cream processing facilities, not the hospital milkshake maker

The whole genome SNP analysis clustered all food, environmental and clinical isolates in 2014 and 2015, except CFSAN028854 (*Asc*I-P4/*Apa*I-P2); and separated them from the outgroup, CFSAN004336, a CC101 strain not associated with the outbreak (Fig. 2). Isolates exhibiting the two clinical PFGE profiles (*Asc*I-P1/*Apa*I-P1 had *Asc*I-P2/*Apa*I-P1) and an isolate exhibiting *Asc*I-P3/*Apa*I-P1 were clustered together (Fig. 2). This is consistent with the epidemiological finding that Company A was the likely source of the outbreak in Hospital X in November 2014. Considering samples from unopened containers of ice cream mixes used to make ice cream/milkshakes in Hospital X in November 2015 were not from Company A and were tested negative for *L. monocytogenes*, and that swab samples throughout the hospital kitchen surfaces were tested negative for *L. monocytogenes* [8], *L. monocytogenes* isolates linked to the illnesses in 2014 likely persisted in the ice cream/milkshake machine of Hospital X through November 2015 and contaminated products that were consumed by the case-patient identified in November 2015. Company B, who purchased ingredients from Company A, yielded an environmental isolate that was clustered together with Company A isolates. Thus, the whole-genome SNP analysis was able to trace the spread of the outbreak strain over more than one facility. A second SNP analysis containing only outbreak-associated isolates identified 59 polymorphic loci and revealed that the pairwise SNP distances among all isolates were 0 to 18 (median of 7). The food, environmental and clinical isolates collected in Hospital X in November 2015 formed a distinct clade (Fig. 2). Two SNPs specifically distinguished the November 2015 isolates from other isolates, one synonymous SNP (nucleotide A in the November 2015 isolates and nucleotide T in other isolates) in an ABC transporter ATP-binding protein (AFY11_ 00690 of the reference genome) and one non-synonymous SNP (nucleotide T in the November 2015 isolates and nucleotide C in other isolates) in 50S ribosomal protein L4 (AFY11_ 15190 of the reference genome).

**Fig. 3:**
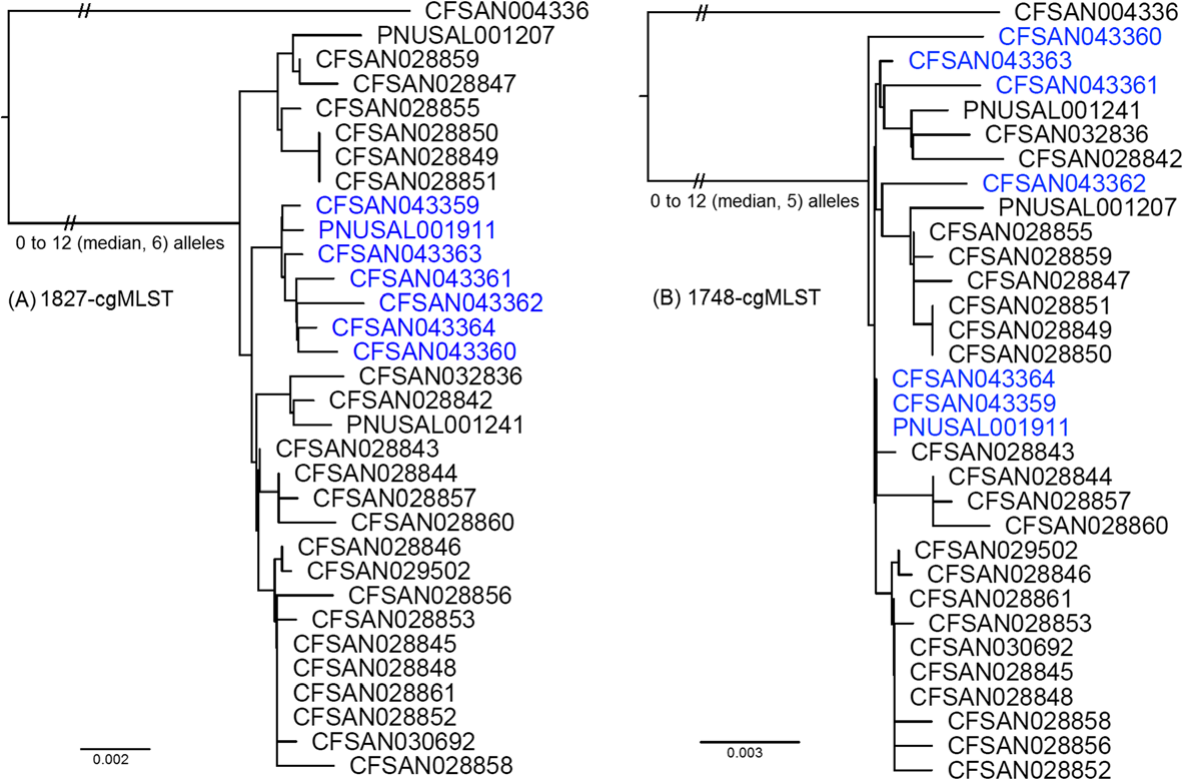
Neighbor-joining phylogeny of outbreak-associated isolates based on two cgMLST schemes, **a** 1827-cgMLST and **b** 1748-cgMLST, using CFSAN004336 for comparison. The trees are rooted at midpoint. The isolates that persisted in the milkshake maker until November 2015 and the clinical isolate collected in November 2015 are in *blue color*. The isolates collected in March and April 2015 were from ice cream processing facilities, not the hospital milkshake maker. The minimum, maximum and median of pairwise allele differences of outbreak isolates are indicated near the root

**Fig. 4:**
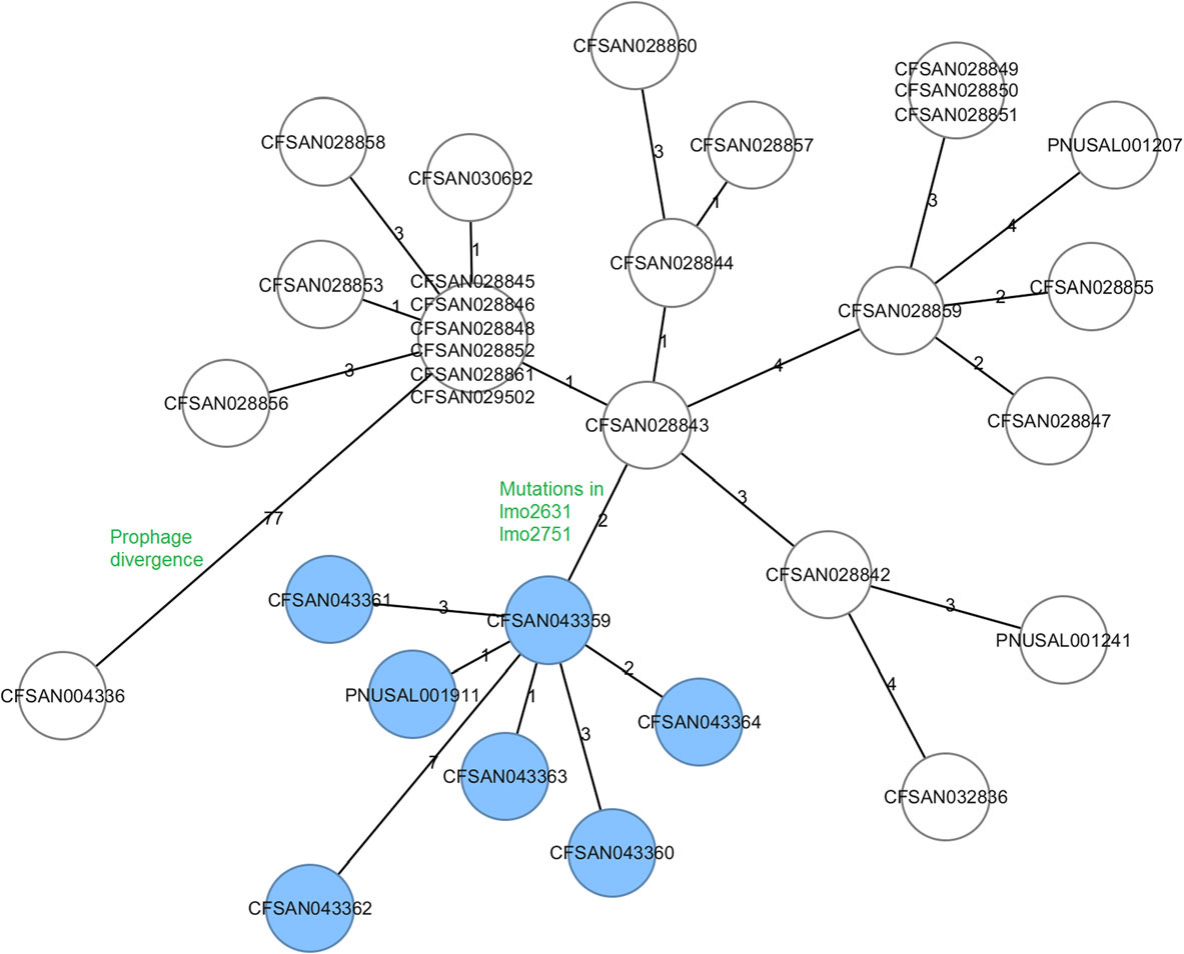
Minimum spanning tree of outbreak-associated isolates based on 1827-cgMLST using CFSAN004336 for comparison. The allele differences between nodes are listed by the connection lines. The isolates collected in November 2015 are marked in *light blue color*. The prophage divergence and SNPs in lmo2631 and lmo2751 are indicated in the figure

A species-specific cgMLST scheme targeting 1827 genes (hereinafter designated as 1827-cgMLST) generated a phylogeny congruent with the SNP-based WGS phylogeny. Outbreak-associated food, environmental and clinical isolates in 2014 and 2015 were clustered together and separated from the outgroup (Fig. 3a). Outbreak isolates differed by 0 to 12 (median, 6) alleles. Among them, isolates collected in 2014 differed by up to 12 alleles; the two 2014 clinical isolates differed by 10 alleles and they differed from the 2015 clinical isolate by 7 and 11 alleles. The isolates that persisted in the hospital milkshake machine until November 2015 and the clinical isolate collected in November 2015 also formed a distinct clade inside the outbreak cluster. Two alleles specifically distinguished the November 2015 isolates from other isolates, lmo2631 (encoding 50S ribosomal protein L4) and lmo2751 (encoding ABC transporter ATP-binding protein). Another species-specific cgMLST scheme targeting 1748 genes (hereinafter designated as 1748-cgMLST) also clustered together outbreak-associated isolates collected in 2014 and 2015 (Fig. 3b). With this scheme, outbreak isolates differed by 0 to 12 (median, 5) alleles. Among them, isolates collected in 2014 differed by up to 10 alleles; the two 2014 clinical isolates differed by 8 alleles and they differed from the 2015 clinical isolate by 4 and 8 alleles. However, the outbreak-associated isolates collected in November 2015 did not form a distinct clade inside the outbreak cluster, because lmo2631 and lmo2751 were not in the gene set targeted by 1748-cgMLST. The minimum spanning tree (Fig. 4) based on 1827-cgMLST also revealed that the November 2015 isolates formed its own clade. This tree does not show a clear central allele profile because the majority of the isolates have their unique allele profiles.

Due to the difference in clustering outbreak-associated isolates collected in November 2015 by the two species-specific cgMLST schemes, we further developed a whole genome-specific MLST scheme (wgsMLST) using the annotated genome (CFSAN028853, GenBank Accession MAKW00000000.1). This scheme targeted 3017 loci in the entire genome of outbreak-associated isolates, which could be alternatively named as outbreak-specific cgMLST because those loci were core to the outbreak isolates. We also performed a previously developed lineage-II specific cgMLST scheme targeting 2342 loci [4]. The results of these two schemes corroborated those of the 1827-cgMLST and whole genome SNP analyses: food, environmental and clinical isolates collected in November 2015 formed a distinct clade inside the outbreak cluster (trees not shown). Both schemes indeed contained the genes encoding the 50S ribosomal protein L4 and the ABC transporter ATP-binding protein.

Two putative complete prophages in the reference genome CFSAN028853 were predicted by PHAST/PHASTER [14, 15]: prophage 1 (31.6Kbp, position 163,457 to 195,099 of contig 11) and prophage 2 (34 Kbp, position 1 to 34,017 of contig 15). BLAST analysis showed that all outbreak isolates contained a conserved prophage 1 (>93% query coverage, >99% sequence identity). BLAST analysis further showed that all outbreak isolates exhibiting *Asc*I-P1 and *Asc*I-P3 contained a conserved prophage 2 (>98% coverage, >99% sequence identity). The outbreak isolates exhibiting *Asc*I-P2 (PNUSAL001241 and CFSAN028861) did not contain prophage 2 (<3% BLAST coverage). The non-outbreak isolate (CFSAN028854) from Company A environment aligned with prophage 1 and prophage 2 for 18% and 71% BLAST coverage, respectively, indicating significant prophage divergence from outbreak isolates. The outgroup CFSAN004336 aligned with prophage 1 and prophage 2 for 87% and 14% BLAST coverage, respectively. The reference genome is a draft genome, and thus multiple fragments that belonged to other putative prophages were also predicted by the software but either they were incomplete prophages or we could not assemble the complete prophages from this draft genome (data not shown). Prophage 2 contained an *Asc*I restriction site (position 9598 to 9605 of contig 15 of reference genome), thus the loss of prophage 2 contributed to the *Asc*I-PFGE pattern change from *Asc*I-P1 or *Asc*I-P3 (all other outbreak isolates) to *Asc*I-P2 (PNUSAL001241 and CFSAN028861). Loss of an *Asc*I restriction site would lead to the combination of two restriction fragments (~185 Kbp and ~250 Kbp) and loss of the prophage 2 would result in a 34 Kbp deletion. Thus the combined fragment should be around 400 Kbp, which was slightly different from the ~430 Kbp fragment (Fig. 1). Thus other DNA variations (e.g., replacement of prophage 2 with a similar-sized prophage through recombination) could co-cause this PFGE banding pattern change. We were not able to identify this variation using the draft genome.

## Discussion

A 7-gene MLST scheme has been used to define major clonal groups of *L. monocytogenes*, designated as CC or singleton [16] and it was recently demonstrated that the this definition was generally compatible with WGS clustering [4, 11]. Isolates associated with this outbreak had ST1038, belonging to CC101 which was commonly isolated in the mid-1950s and its prevalence had since decreased [17]. However, it likely has re-emerged recently, evidenced by its isolation in 31 clinical cases in Lombardy, Italy between 2006 and 2010 [17], as well as its association with a 2012 U.S. outbreak linked to ricotta salata cheese products manufactured in Italy [18]. Thus, CC101 has been involved in at least two outbreaks to date and represents an epidemic clone. *inlA* encodes internalin A, which is involved in the invasion of human intestinal epithelia cells and could play an important role in *Listeria* virulence [19]. Premature stop codons (PMSCs) in *inlA* lead to truncated protein in some *Listeria* strains and were linked to attenuated virulence of those strains in mammalian hosts and thus it has been [19]. Moreover, PMSCs are mostly found in lineage II strains and isolated more frequently from food and food production environment than from human listeriosis cases [19]. Our finding is consistent with previous observation that outbreak-associated strains generally do not contain PMSCs [20]. To date, 4 *Listeria* pathogenicity islands (LIPI) have been characterized as virulence factors: LIPI-1 is conserved across the entire species of *L. monocytogenes* [21]; LIPI-2 is specific to *L. ivanovii* [22]; LIPI-3 is mostly present in lineage I isolates [23]; and the very recently identified LIPI-4 is present in several lineage I CCs and a few lineage II, III and IV strains [11]. This explains why we observed only LIPI-1 in isolates associated with this outbreak. In comparison, the 2010–2015 multistate ice cream outbreak strains (CC5) contained LIPI-1, but not LIPI-3 or LIPI-4 [6] and the recent isolates (singleton ST382) from the stone fruit, caramel apple and leafy green salad outbreaks contained all LIPI-1, LIPI-3 and LIPI-4 [24].

In this study, WGS could not only trace the transmission of the outbreak strain between two facilities, but also reveal its persistence in the soft serve ice cream/milkshake machine for one year. Furthermore, WGS was able to cluster all outbreak-associated isolates, despite their difference in *Asc*I-PFGE profiles. The milkshake maker had a regular sanitation schedule, and extra sanitizing effort after the outbreak, including disassembly of the machine at least twice, was taken after the outbreak recognition [8]. Thus, it is probable that while the majority of the *L. monocytogenes* cells contaminating the milkshake maker in 2014 were eliminated during sampling and sanitizing; a few cells escaped and/or survived, contaminated other areas of the milkshake maker later, and eventually made their presence in the final product during operation. This also concurred with our results that the food, environmental and clinical isolates collected in November 2015 formed its own WGS clade and thus had a recent common ancestor.

The high resolution of WGS has been demonstrated in various studies by differentiating outbreak isolates from non-outbreak isolates, especially those matched by PFGE [5, 6]. In addition, WGS can achieve more than just discrimination between unrelated isolates. For example, it can be used to study the microevolution of different isolates in the same outbreak setting, identify genotypes that may be specific to different product varieties or production lines in the same facility [6], and ultimately shed some light on root cause analysis. For another example, combination of core genome and accessory genome was needed to elucidate the epidemiology of isolates persistent in a food processing facility for 12 years [4]. Comparison between outbreak and non-outbreak isolates could provide potential candidates for future functional genomics analyses on virulence. Comparison among strains persistent in an environment could identify potential candidates for studying the evolution and mechanisms of *L. monocytogenes* persistence. WGS analyses targeting the entire genome could certainly reveal more potential genetic markers. For example, in this study, whole genome-based analyses, lineage-specific cgMLST and 1827-cgMLST identified unique variants in the genes encoding ABC transporter ATP-binding protein (lmo2751 in EGD-e) and 50S ribosomal protein L4 (lmo2631 in EGD-e) of the persistent cells, which contributed to the formation of the distinct clade containing these persistent cells in the phylogenetic trees; while 1748-cgMLST did not yield that clustering because of the absence of these two genes in its gene set. The 1827-cgMLST scheme and the 1748-cgMLST scheme share 1324 common loci (i.e., there are 503 unique loci in the 1827-cgMLST scheme and 424 in the 1748-cgMLST scheme), thus, despite their sufficient discriminatory power to distinguish epidemiologically unrelated isolates, differences in results are expected when they are applied to study the microevolution within the scope of an outbreak. We also evaluated another cgMLST scheme targeting 1701 core genes of *L. monocytogenes* [12] which contained lmo2751 and lmo2631, and it generated a phylogeny congruent with that by 1827-cgMLST and SNP analyses: the November 2015 isolates formed a distinct clade within the outbreak cluster (tree not shown). It is possible that different isolates collected in November 2015 simultaneously accumulated SNPs in both genes during evolution from their 2014 ancestor; however, it is also possible that their 2014 ancestor already had these two unique SNPs, which remained unchanged during persistence in the milkshake maker until November 2015. The expression of 50S ribosomal protein L4 in *L. monocytogenes* is affected by alkali-tolerance response, which may be critical for this pathogen to survive in human gastrointestinal tract and during food processing [25]. Mutation in this gene has been linked to antimicrobial resistance in other bacteria [26, 27]. The ABC transporter ATP-binding protein encoded by lmo2751 is upregulated when *L. monocytogenes* was exposed to bacteriocin pediocin [28] or during growth within murine macrophages [29]. The exact roles of these two proteins in the survival and evolution of *L. monocytogenes* in food processing environment remain to be investigated.

Generally, a cgMLST scheme targets the entire population of *L. monocytogenes* [4, 11–13] and the core loci comprise ~60% of a typical coding genome. The species-specific schemes are suitable for evolutionary analysis and nomenclature. A standardized nomenclature system based on cgMLST could be beneficial in surveillance studies because isolates from different environment, food commodities and geographical locations analyzed in different studies can be easily compared to suggest possible links. However, vigorous collaborative validation needs to be performed on multiple elements in this nomenclature system: the centralized database, the set of cgMLST targets, the platform(s) to run the analysis, the parameter(s) and algorithm to call allele differences, the mechanism to deal with missing regions due to draft sequencing, and the threshold to define cgMLST or cluster types. Minimum spanning tree based on a 7-gene MLST has been used to define clonal complexes [16], in which the majority of the strains share the same sequence type, and that sequence type serves as the central allele profile to define single locus variants. This approach might not be suitable for cgMLST since isolates could easily differ by one allele in cgMLST and thus no clear central allele profile can be easily identified, as illustrated in this outbreak (Fig. 4). An approach to define cgMLST cluster type is to set an allele threshold among isolates, but different studies have proposed different thresholds [4, 11, 12]. In addition, such approach is not perfect because the entry order of submission of a set of isolates could potentially affect the assignment of cgMLST cluster types of those isolates [30]. Nonetheless, species-specific cgMLST schemes are generally satisfactory in differentiating epidemiologically unrelated isolates [4, 11, 12]. However, when WGS is used to differentiate among a set of closely related isolates and/or to study the microevolution of isolates within the scope of one outbreak, a flexible definition of cgMLST could be explored to fully utilize the high resolution of WGS [31]. cgMLST schemes specific to individual lineages of *L. monocytogenes* contain loci comprising ~80% of a typical coding genome [4]. In this study, we developed a wgsMLST, which was essentially an outbreak-specific cgMLST, to analyze the microevolution among outbreak isolates. In contrast to wgMLST which contains a pre-defined set of pan-genome loci [10], this wgsMLST targets the entire set of coding loci of an outbreak isolate, and thus could target any novel loci that are not in the pan-genome pre-defined based on a set of previously published genomes. This is similar to the whole genome SNP-based approach that maximizes the resolution by selecting one outbreak isolate as the reference to analyze other outbreak isolates [32].

Several previous studies using SNP-based WGS analysis employed a complete genome closed by PacBio® technology as the reference [5, 24]. However, using PacBio® may not be practical in every outbreak investigations. Here, we explored the use of a CLC Genomics Workbench-assembled draft genome (CFSAN028853) as the reference for the CFSAN SNP Pipeline and produced a WGS phylogeny that supported PFGE and epidemiological evidence. We also tried CFSAN028853 assembled by SPAdes assembler 3.9.0 [33], and the WGS analysis generated the same phylogeny with minor changes of the lengths of several tree branches (data not shown). CLC and SPAdes both map raw reads to initial assembly for error correction; however, error in the final assembly could still occur. We mapped raw reads of CFSAN028853 to both CLC assembly and SPAdes assembly using the CFSAN SNP Pipeline and found that raw reads were consistent with CLC assembly but differed from the SPAdes assembly by 6 SNPs. As a result, between the final SNP matrices generated using CLC and Spades assemblies the pairwise SNP differences of several isolate pairs differed by 1–2 SNPs. Thus, we believe the CLC assembly was more accurate than the SPAdes assembly in this case, although we found that SPAdes can be more accurate in other cases (data not shown). Nonetheless, using either assembly as the reference yielded the same WGS clustering. The wgsMLST was defined using the annotated, CLC-assembled draft genome of CFSAN028853. A completely closed CFSAN028853 genome could probably have revealed additional coding regions as wgsMLST loci and thus further improved the resolution of wgsMLST, although this improved resolution may not be critical for the purpose of outbreak investigation.

*Asc*I-PFGE banding pattern changes due to prophage variations have been observed among isolates associated with a few outbreaks. In this outbreak, a prophage loss led to the loss of an *Asc*I restriction site in the prophage, resulting in combination of two *Asc*I fragments, although other unidentified DNA variations could co-affect the PFGE banding pattern change from *Asc*I-P1/*Asc*I-P3 to *Asc*I-P2. DNA variations underlying the difference between *Asc*I-P1 and *Asc*I-P3 were not identified. Among isolates of several other outbreaks, gain/loss of prophage in a PFGE fragment caused the fragment to shift to a different position in the gel [6, 34, 35]. In one outbreak, gain/loss of 3 prophages occurred among different isolates, and *Asc*I restriction analysis of the completely closed genome allowed the precise determination of the genome positions of all *Asc*I fragments, which unambiguously identified the gain/loss of the specific prophage in the specific *Asc*I fragment [6]. Previous analyses have shown that reference-based reads mapping and SNP calling in repetitive regions and insertion regions of prophages could yield inconclusive SNPs, which were usually present in high density (≥3 SNPs in 1000 bp) [6, 24]. Recombination events could also generate high - density SNPs [6]. Thus, when analyzing a group of closely related isolates (e.g., ≤50 SNPs in 3 × 10^6^ bp) associated with the same outbreak, CFSAN SNP Pipeline offers the option to apply a filter to remove these high-density SNPs from the final SNP matrices. In this study, the 4 removed high - density variant regions contained SNPs only between PNUSAL001241/CFSAN028861 and other isolates, and these high - density variant regions were all in insertion sites of putative prophages: the prophage 2 described above, and 3 fragments from other incomplete prophage(s) (data not shown).

The patients in Hospital X involved in the Washington State outbreak under discussion in this study and patients in a hospital (Hospital Y) involved in the 2010–2015 multistate outbreak [6] consumed milkshakes prepared from contaminated ice cream products, and this highlights the potential of ice cream as a vehicle for listeriosis infection, given that *L. monocytogenes* could grow in milkshakes, especially when the milkshakes go through temperature abuse during serving. However, *L. monocytogenes* in milkshakes prepared from the multistate outbreak-associated ice cream had a relatively long log phase (9 h) and a slow growth rate of 0.186 CFU/log/h at room temperature, which could be attributed to low level of initial amount of naturally occurring *L. monocytogenes* [36] and/or the variety and levels of competing microflora present in the ice cream samples [37]. The ice cream products associated with the Washington State outbreak were not available for such analysis. The milkshake maker used in Hospital Y involved in the multistate outbreak was a drink mixer employing simple propellers and were relatively easy to clean [36], and the environmental testing in the hospital kitchen, including the drink mixer, did not yield *L. monocytogenes* [38]. The milkshake maker used in Hospital X was a soft serve shake freezer which held ice cream mix and made milkshake inside the machine to serve through the dispensing nozzle, and had reusable parts of reservoirs, pipes and mixers that made contact with ice cream [8]; thus it was more difficult to clean, which could explain why *L. monocytogenes* was able to survive the sampling and cleaning. The occurrence of these two outbreaks, involving patients with weakened immune systems, could contribute to our understanding of the risk associated with *L. monocytogenes* contamination in ice cream.

## Conclusions

WGS analyses clustered epidemiologically related isolates, and clarified the microevolution and persistence of isolates within the scope of one outbreak. A flexible definition of core genome MLST, targeting a species, a genetic lineage or an outbreak, could be explored to offer different levels of resolution based on the set of strains investigated and the purpose of the analysis.

## Methods

### Isolates

*L. monocytogenes* isolates were collected from ice cream products and environmental samples from Company A as well as patients and ice cream products from Hospital X during the outbreak investigation in 2014. An environmental isolate from Company A and an environmental isolate from Company B were collected in early 2015 as Company B purchased dairy ingredients from Company A [7, 8]. Isolates were also collected in November 2015 from the patient, the ice cream that remained in and were dispensed from the milkshake maker in Hospital X, and the environmental samples from different areas (e.g., side walls of internal parts and nozzle assembly) of the milkshake maker. Sequences are publicly available at NCBI Sequence Read Archive (SRA) of the GenomeTrakr database [39] with SRA ID listed in Table 1.

### Whole genome SNP analysis

WGS was performed using Illumina MiSeq platform (Illumina, San Diego, USA) as previously described [40]. Reference-based whole genome SNP analysis was performed using SNP pipeline (version 0.7.0) developed by the Center for Food Safety and Applied Nutrition (CFSAN), as previously described [32, 40, 41]. CLC Genomics Workbench 9.0.1 (Qiagen, Hilden, Germany) was used to assemble CFSAN028853 (200× coverage, GenBank Accession MAKW00000000.1) to serve as the reference. A *L. monocytogenes* ST101 strain, CFSAN004336 (Table 1), which belonged to the same CC101 as the outbreak-associated isolates, was used as the outgroup to demonstrate that WGS can separate this strain from the outbreak-associated isolates. A second WGS analysis was performed on only the outbreak-associated isolates for precise SNP calling because accurate SNP calling by reference-based methods may be affected by ascertainment bias when these methods were applied to slightly more diverse isolates [42, 43]. Briefly, raw reads from each isolate were mapped to the reference genome using default settings of Bowtie2 version 2.2.9 [44]. The BAM file was sorted using Samtools version 1.3.1 [45], and a pileup file for each isolate was produced. These files were then processed using VarScan2 version 2.3.9 [46] to identify high quality variant sites using the mpileup2snp option. A Python script was used to parse the.vcf files and construct an initial SNP matrix. For these closely related isolates, the SNP Pipeline applied a filter to exclude variant sites in high density variant regions (≥3 variant sites in ≤1000 bp of any one genome) since they may be the result of recombination or low quality sequencing/mapping and/or be associated with repetitive elements. Four regions, 42 bp (containing 16 variant sites), 387 bp (14 variant sites), 95 bp (4 variant sites) and 102 bp (9 variant sites), all in prophage insertion areas, were filtered out. The 9 variant sites in the 102 bp region were also within 500 bp of the start of a reference genome contig, which typically had lower quality of mapping and assembly due to less reads mapped to contig ends. GARLI was used to create topologies based on the SNP matrix [47].

### Species-specific and lineage II-specific cgMLST analyses

Two species-specific cgMLST schemes, developed using strain EGD-e (GenBank Accession NC_003210.1) as the reference, were used to analyze the isolates: the 1827-cgMLST targeting 1827 core genes [4] and the 1748-cgMLST targeting 1748 core genes [11]. Core genome loci of both schemes were incorporated into Ridom SeqSphere + (Ridom GmbH, Münster, Germany) and cgMLST was performed using default settings as previously described [4]. Neighbor-joining and minimum spanning trees were constructed from the allele profiles of all the isolates. A previously developed lineage II-specific cgMLST, targeting 2342 core genes of lineage II, was also performed [4].

### wgsMLST/outbreak-specific cgMLST

The reference genome used for SNP analysis, CFSAN028853 (GenBank Accession MAKW00000000.1), was used as the only genome to define a wgsMLST scheme using the cgMLST target definer (version 3.5.0) function of SeqSphere^+^ (Ridom GmbH, Germany) with default parameters as previously described [12]. The software collected all coding loci of CFSAN028853 and filtered out those that could be generated by assembly/annotation errors, resulting in 3107 loci. These loci were core to the outbreak isolates and thus could be alternatively called as an outbreak-specific cgMLST.

### In silico MLST, prophage, virulence gene presence and *inlA* premature stop codon analyses

In silico MLST analysis was performed using the tools in the BIGSdb-Lm database (http://bigsdb.pasteur.fr/listeria/listeria.html). Putative prophages were identified from individual contigs of the reference genome (CFSAN028853) by PHAST/PHASTER [14, 15], and the predicted complete phages were compared to other isolates using BLAST. A threshold of ≥60% query coverage with ≥80% sequence identity [48] of BLAST alignment indicated the presence of a CFSAN028853 prophage in a genome. The presence of major virulence genes [11] was examined using BLAST. The *inlA* sequences of all isolates were examined for the presence of premature stop codons.

### Pulsed-field gel electrophoresis (PFGE)

PFGE was performed using the PulseNet standard protocol [49].

## Abbreviations

CC: Clonal complex
cgMLST: Core genome multilocus sequence typing
PFGE: Pulsed-field gel electrophoresis
PMSC: Premature stop codon
SNP: Single nucleotide polymorphism
ST: Sequence type
WGS: Whole genome sequencing
wgsMLST: Whole genome-specific multilocus sequence typing
